# Supplementary material for: Formoterol Acting via β2-Adrenoreceptor Restores Mitochondrial Dysfunction Caused by Parkinson’s Disease-Related UQCRC1 Mutation and Improves Mitochondrial Homeostasis Including Dynamic and Transport
Source: Biology (Basel). 2024 Mar 30;13(4):231. doi: 10.3390/biology13040231 (PMC11048601; doi:10.3390/biology13040231)
Supplement: Supplementary file 1 [file biology-13-00231-s001.zip › Supplements-R.pdf]

Table S1. Distinct differences in expression of phosphorylated proteins among the compared sets of groups. Up- and down-regulation of corresponding proteins by formoterol treatment were shown as green color and blue color, respectively.

Inter-groups	UQCRC1^{mut}-DMSO vs. WT	UQCRC1^{mut}- F vs. -DMSO	UQCRC1^{mut}- F+P vs. -P
Items	Fold change %	Fold change %	Fold change %
4E-BP1-Thr36	-91.903	181.746	-26.913
ERK-1-T202/Y204	-33.135	86.279	-14.374
ERK-2-Y185/Y187	-39.550	114.840	-18.364
GSK3a-Ser21	-30.962	131.014	-38.359
GSK3b-Ser9	-1.754	21.504	-14.164
PRAS40-Thr246	-40.695	69.764	34.059
Raf-1-Ser301	-11.214	64.352	-18.239
RPS6-Ser235/236	-12.353	28.866	2.270
BAD-Ser112	-26.780	2.590	18.443
PTEN-Ser380	4.123	5.024	-3.928
RSK2-Ser383	-7.614	-25.871	40.404
PDK1-Ser241	4.528	3.495	-5.904
P53-Ser15	3.967	2.819	-7.564
mTOR-Ser2448	1.201	0.937	17.016
AKT-Ser473	39.467	-32.596	24.209
RSK1-Ser380	10.311	-11.411	10.429
P70S6K-Thr421/Ser424	6.247	-18.528	0.293
p27-Thr198	10.586	-0.689	5.904

Figure S1. Cell viability of wild-type (WT) and mutant UQCRC1 knock-in human neuroblastoma SH-SY5Y cells exposed to various doses of formoterol or propranolol for 24 hours. Cell viability was assessed by the WST-1 assay. **P* < 0.05 vs. the DMSO group. Data are presented as the mean ± SD. N=5

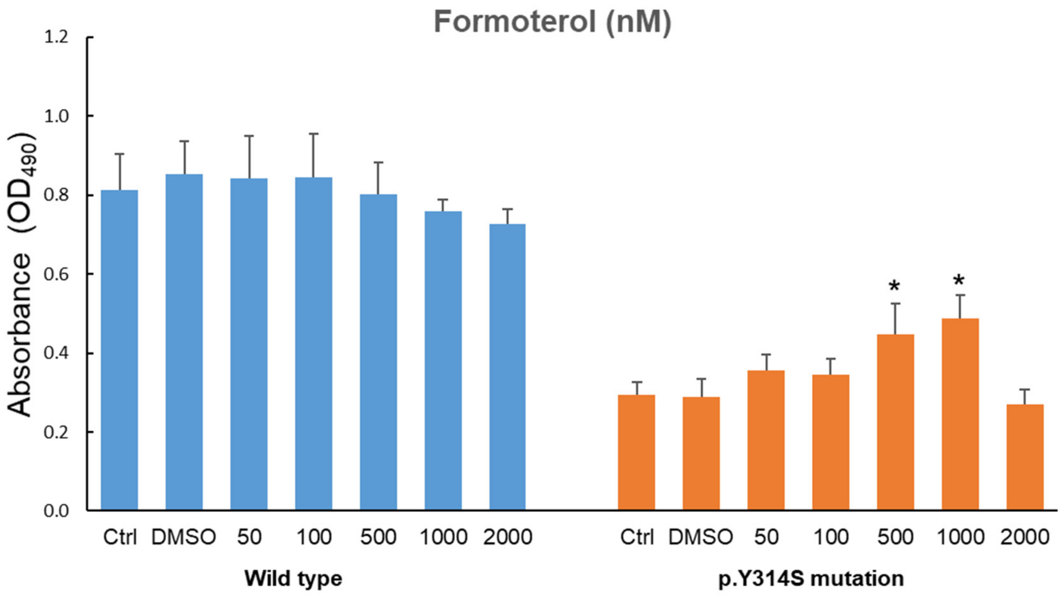

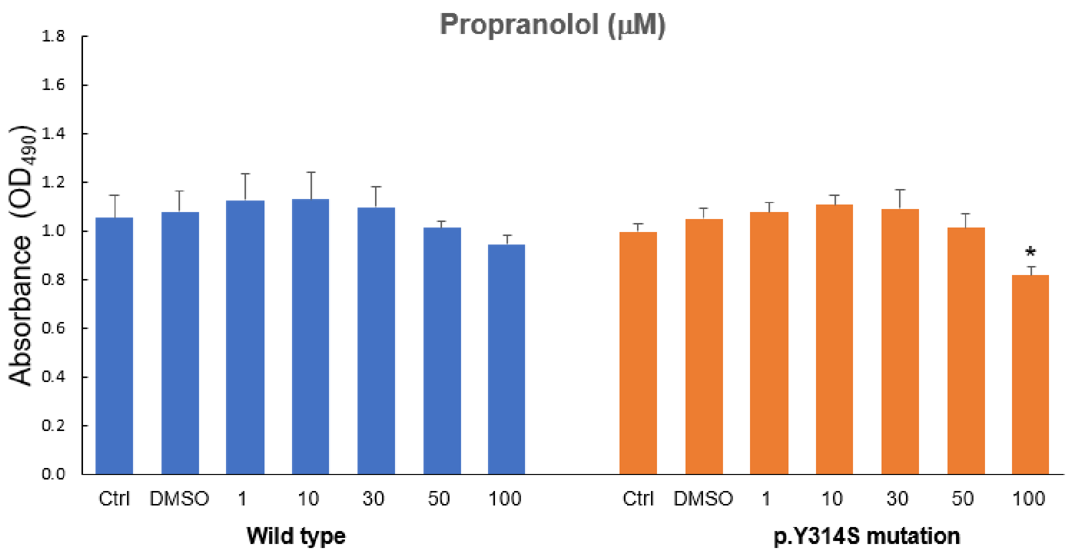

Video S1: Movement of MitoGFP-labelled mitochondria in wild-type cells.

Video S2: Movement of MitoGFP-labeled mitochondria in DMSO-treated UQCRC1-mutant cells.

Video S3: Movement of MitoGFP-labeled mitochondria in formoterol-treated UQCRC1-mutant cells.

Video S4: Movement of MitoGFP-labeled mitochondria in formoterol plus Propranolol-treated UQCRC1-mutant cells.
